# A quality improvement project of patient perception of AI-generated discharge summaries: a comparison with doctor-written summaries

**DOI:** 10.1308/rcsann.2025.0014

**Published:** 2025-04-03

**Authors:** J Bass, C Bodimeade, N Choudhury

**Affiliations:** Surrey and Sussex Healthcare NHS Trust, UK

**Keywords:** Artificial intelligence, Discharge, Tonsillectomies

## Abstract

**Introduction:**

Every patient admitted to hospital should receive a discharge letter when they leave. Artificial intelligence (AI) has the capability to fulfil this task. Here, we investigate the use of AI to generate discharge letters compared with letters written by a doctor.

**Methods:**

Using an AI tool, ChatGPT, we generated two discharge letters for hypothetical elective tonsillectomy patients. We asked the parents of paediatric tonsillectomy patients to blindly compare the AI letters with two anonymised real discharge letters for tonsillectomy patients, written by two ear, nose and throat (ENT) doctors. Participants were asked to rate the quality of medical information, the ease of reading and the length of each of the four discharge letters. They were also asked to deduce who they thought wrote each discharge letter (AI or a doctor).

**Results:**

Forty-seven parents responded to the survey. Our results demonstrate that the AI letters were reported to contain significantly better medical information (*p* = 0.0059) and were significantly easier to read than the doctor-written letters (*p* < 0.0001). Respondents had a 50% sensitivity in correctly identifying the letters written by AI.

**Conclusions:**

AI tools have the potential to write tonsillectomy discharge letters of comparable quality (as perceived by our participant population) to those written by ENT doctors. This study provides preliminary evidence to show that AI-generated discharge letters may be an interesting avenue of further investigation as an application for this tool.

## Introduction

Artificial intelligence (AI) is increasingly present in different sectors, and healthcare is no exception. The focus of developing AI in medicine has been to aid our ability to treat patients, whether that be in its use in the development of new medication regimens, interpreting radiological imaging or in assisting surgeons in decision making during an operation.^1^

There has been some research exploring the opinions of healthcare professionals and patients on the use of AI in healthcare, discussing future applications, their trust in AI-driven clinical decisions and their understanding of AI.^2–9^ There has been evidence of discomfort from patients in these studies of using AI in lieu of a human doctor. Therefore, the use of AI requires evidence that it is more reliable than its human counterpart, because this would then lead to developing trust in AI use in the healthcare setting. One of the most basic and common tasks of a day one doctor in training is creating discharge letters. The objective of this quality improvement project was to compare the quality of an AI-generated discharge summary with a doctor-generated discharge summary, as judged by the parents of paediatric patients. The AI platform used for this study is the frequently used ChatGPT developed by OpenAI.^10^

## Methods

### Reporting guidelines

This quality improvement project has been reported according to the CHERRIES guidelines.^11^

### Quality improvement project registration

This project was registered with Surrey and Sussex Healthcare NHS Trust's audit department.

### Ethics

In accordance with the Medical Research Council's guidelines for health research, ethical review was not sought for this study. The Health Research Authority decision tool, used to assess the necessity of ethical approval, indicated that this study fell outside the scope of requiring formal ethical review.^12^

### Generating the discharge summaries

Four separate discharge summaries were compared. Two discharge letters were written by two doctors in autumn and winter 2022 for real paediatric tonsillectomy patients, whose personal information was removed. Two other letters were written for fictitious paediatric tonsillectomy patients by the AI tool Chat GPT (OpenAI).^13^ In March 2023 the following prompt was issued to Chat GPT 3.5 (the most powerful OpenAI freely available at the time): “Please can you write a discharge letter for a patient who has had a bilateral tonsillectomy surgery including after care advice.” This is in keeping with the type of instruction a day one doctor would be given by their seniors. This command was repeated three times, then one of the letters generated was chosen before they were read. The method of randomisation involved rolling a dice, with numbers 1 and 4, 2 and 5, and 3 and 6 relating to letters A, B and C, respectively. Letters A and C were selected and used in the study. The letters were checked by three members of the team, all of whom are qualified doctors, to ensure there was no misleading or incorrect advice.

### Participant population

There were 47 respondents in this survey. The respondents were parents of paediatric patients who underwent elective tonsillectomy surgery as a day-case procedure in a district general hospital in the United Kingdom between June 2023 and June 2024. The respondents were informed of the goals of the project, and advised that if they chose not to be involved it would have no impact on the patient care received and that their personal information would not be stored or used after their participation. There was no assessment or exclusion of participants based on patient demographics, education level or employment status, thus attempting to keep the patient population representative of the local population. Participants were asked to complete a questionnaire while waiting for their child to be discharged home during the routine postoperative observation period. All the patients received a separate discharge summary written by a doctor from their surgical team and were advised to follow the advice from that summary. All participants gave verbal consent for their responses to be used anonymously. Respondents were asked to input their email address when answering the survey, to ensure duplicate responses could not be filled in by the same person. There were no partial responses.

### Assessing quality of discharge summaries

Using an online questionnaire tool (Google Forms), a survey was created to assess the quality of the four discharge summaries.^14^ This had been tested on colleagues to ensure it was functional and easy to use. The respondents were blinded to which letter was generated by ChatGPT or by an ENT doctor.

Participants were asked to assess three domains of the discharge summaries by answering the following questions:

- quality of the medical information
- ease of reading
- length of letter.

They were also asked to judge whether they believed the summary they read for each of the four discharge summaries was written by AI or a human doctor. The quality of medical information was not measured using a validated tool; instead we used the participants' perception of quality. Likert scales were used to compare the discharge summaries, and the anchors are demonstrated in Table 1.

**Table 1:** Likert scale anchors and ideal scores for each survey question

Survey question	Low anchor	High anchor	Ideal score
Quality of medical information	1 = Very Poor	5 = Excellent	5
Ease of reading	1 = Very Poor	5 = Very Easy	5
Length of letter	1 = Too Short	5 = Too Long	3

**Table 2:** Depicting the mean scores of areas compared between artificial intelligence (AI)-generated discharge summaries and doctor-written discharge summaries

	Quality of medical information	Ease of reading	Length of letter	Author correctly identified by respondents (%)
AI	3.87	4.37	3.13	50.00
Human	3.44	3.72	2.98	47.87
*p*-value	0.0059*	<0.0001*	0.1952	0.6712

*Statistically significant.

No real patients received a discharge summary written by ChatGPT.

### Statistical analysis

To assess the statistical significance of the results of quality of medical information, ease of reading and length of letter, a paired *t*-test was used to generate *p*-values. To assess the statistical significance of the difference in ability to identify the correct author between the two groups, a chi-squared calculation was used. Statistical analyses were completed using Graph Pad Prism 10 software packages. Results were deemed statistically significant if *p* < 0.05.

## Results

Letters A and C were written by ChatGPT, whereas letters B and D were written by an ENT doctor. Participants assessed discharge letter quality in three domains: quality of medical information, ease of reading and length of letter. Averages of the scores were used to determine which of the four letters was of better quality in each domain (summarised in Table 2).

Forty-seven participants responded to the survey. The AI letters’ average score was 3.87 for quality of medical information compared with an average score of 3.44 for doctor-written discharge summaries, suggesting that AI discharge letters contained better medical information (*p* = 0.0142). AI-generated letters’ average scores for length of letter and ease of reading were 3.13 and 4.37, respectively, and the AI-generated letters were considered to be significantly easier to read than the doctor-written letters (*p* < 0.0001). Respondents had a 50% sensitivity in correctly identifying the letters written by AI, and a 51.14% sensitivity in correctly identifying letters written by doctors. There was no statistically significant difference in ease of identifying the author between the two groups (*p* = 0.671). AI-generated letters received a higher mean rating for length of letters; however, the difference in means between the groups was not statistically significant (*p* = 0.1952).

## Discussion

### Summary

This quality improvement project was intended as a pilot study to demonstrate that simulated discharge summaries, generated by ChatGPT 3.5 using a generic prompt, can contain higher quality medical information than summaries written by an ENT doctor using real cases (as perceived by our participant population), and provide evidence that this is a useful avenue of further research. Furthermore, 50% of respondents in this study deduced correctly that letters A and C were written by AI, whereas 47.87% of respondents could identify letters B and D were written by doctors. This difference was not statistically significant (*p* = 0.6712) indicating that the respondents in this study were unable to confidently discern between AI-generated discharge summaries and doctor-written discharge summaries, no matter who wrote the summary.

### Study strengths and limitations

The strength of this study is that we had a sufficient sample size of respondents from the parents of patients to enable appropriate statistical analysis of our results.

Our results allow us to only confidently conclude our findings based on the study cohort. Our participant population perceived the discharge summaries written by ChatGPT 3.5 to be of higher quality and easier to read than the human counterpart summary for a paediatric patient undergoing elective bilateral tonsillectomy surgery. Although large numbers of patients, especially children, have similar medical histories and undergo the same routine procedure, there is of course variation in both patients and surgical techniques used, even for this operation. Our results may therefore not be applicable in more complex surgical cases or where the patient cohort may be more complex. Our participant population was limited to patients only, and we recognise that surveying other stakeholders, such as general practitioners (GPs), pharmacists, nurses or specialty doctors, would allow us to develop a broader picture of whether AI-generated surveys are a useful avenue of exploration.

Furthermore, the discharge summaries reviewed here were compared with only two summaries written by two doctors. As such, extrapolating our findings beyond the clinical context of a healthy paediatric patient undergoing a tonsillectomy would need to be carefully considered.

At the time of the conception of this project, ChatGPT 3.5 was the most widely available and user-friendly large language model (LLM) suitable for the authors' purposes. However, it is important to note that ChatGPT 3.5 is not intended, nor is it registered, as a medical device and therefore cannot be applied directly to medical practice. Since the conclusion of this study, newer and potentially more appropriate LLMs may now be available, which could be better suited for similar applications.

### AI in the literature

A recent qualitative study explored the opinions of 24 experts on how AI is likely to transform medical care, and one of the conclusions was that the inability of AI to imitate human connection will limit its application to other tasks which, at present, are carried out by doctors.^9^

However, given that we can potentially augment a doctor’s productivity by utilising AI to help with some tasks, this study provides evidence that using AI does not compromise care and can be used to help complete administrative tasks. This may need to be done with some caution and doctor oversight, to allow for variations in patient or surgical factors, where this may potentially require more tailored advice in the discharge summary, as well as ensuring these tools are used within the correct regulatory frameworks.

Clough *et al* explored the use of AI to produce discharge summaries, comparing the quality with that of summaries written by clinicians.^15^ These were judged by GPs, who decided whether each of the summaries was acceptable or not, concluding that the AI could produce summaries of sufficient quality. The GPs were also blinded to the authors, and asked who they thought wrote each summary, with a sensitivity of 52%, which was similar to our respondent accuracy (49%). Our study has similar conclusions, that quality is at the very least preserved. Although AI has great promise in healthcare, there are also privacy concerns, variability in its output, AI hallucination and the need for clear regulatory frameworks and enforcement, as well as solutions to the challenge of licensing LLMs as medical devices.^16^

### Implications of research and practice

There are several steps to ensuring a smooth transition from secondary care back to primary care, and there are therefore multiple opportunities for inaccuracies or errors. This starts with documenting the appropriate information on the discharge summary, prescribing medications correctly and documenting any changes made to the medications, as well as ensuring appropriate investigations and follow-up are organised.

Discharge summaries play an especially significant role in the communication of ongoing care between the admitting team, the patient and their GP. Poor quality discharge summaries can lead to patients not receiving adequate safety-netting advice, missed pending results or missed follow-up clinic appointments, and lead to a loss of faith between patient and healthcare providers, as well as leaving GPs in the dark about the care their patients received, and changes made to long-term plans. At present, there is evidence that patients receive discharge summaries without key components, affecting ongoing patient care.^17^ There is also evidence in the literature that a substantial number of discharge summaries have prescription errors.^18^

A limiting factor to efficient high-quality care is the availability of clinician time. There is increasing demand on public health services, longer waiting times in emergency departments and more patients staying in hospital for more than 12h.^19^ This subsequently puts more pressure on doctors in training to improve ‘flow’ in a hospital by discharging patients quickly, and subsequently documentation length and quality can decrease.

An observational study exploring how much time surgeons dedicate to different tasks throughout the day found that, on average, surgeons spent a mean of 1h 25min documenting for their patients, and a further 46min a day on general administrative tasks. The mean length of time surgeons spent performing or assisting in surgery was 2h 20min. Job satisfaction with different daily tasks was assessed, and the mean scores (out of 5) for documentation and administration were 2.96 and 2.63, respectively.^20^ One of the largest contributors to physician burnout is workload, including inefficient work processes (such as extensive documentation), and tasks not considered meaningful to physician work-related activities.^21^

Given the impacts of clinician burnout on both patient care (increased medical errors, drop in quality, longer recovery times and patient satisfaction) and the clinician (increased depression, anxiety, poor self-care, increase in substance abuse), tools to improve factors that increase the likelihood of burnout, such as a high workload, should be utilised.^21^

As of November 2023, 90% of National Health Service trusts have electronic patient records.^22^ As AI technology develops further, incorporation of AI into existing IT systems will allow up-to-date information for the generation of documentation, including discharge summaries.

Incorporating AI into healthcare provision will be essential to improve quality of care and the efficiency of healthcare providers. Its use in administrative tasks will allow skilled workers to focus on other tasks and reduce workload burden. Its incorporation will need robust regulation and a review of the tasks it is used for. One priority will be to ensure that its performance is continually validated, and AI use is not causing harm by having insufficient safeguards against AI hallucination. It must be General Data Protection Regulation compliant, and patients fully informed of the nature of AI involvement in their care, and how AI uses their data. It must have sufficient human oversight, and its use should support the removal of health disparities, not deepen them as experienced by vulnerable groups.^22^ This study was not designed to legitimise a certain type of software for wider application, but rather to explore how a discharge summary created using a widely available LLM, when given a prompt that would be expected to be given to a day one doctor, compares with a doctor-written summary. It provides evidence that this is an interesting area for further investigation, and as AI tools become more tailored, and the regulatory frameworks and safeguards are put in place, it would be interesting to see whether our results are replicated in these settings. Another area of interest would be to replicate this methodology with different stakeholders, such as GPs, specialty doctors, pharmacists, nurses and other members of the multidisciplinary team.

## Conclusions

Our results demonstrate that, under these specific conditions, AI-generated discharge summaries are of comparable, if not better, quality, readability and length than doctor-written discharge summaries, as perceived by our patient population. In our cohort respondents were unable to accurately distinguish between AI-generated discharge summaries and human-generated discharge summaries.

The results of this study highlight a potential area of using AI for administrative tasks which will in turn free-up clinicians to perform more clinically oriented tasks and reduce workload.

AI should not replace human judgement or expertise, but rather augment and assist doctors in delivering high-quality and patient-centred care. Other interesting areas of investigation on this topic include informed consent for this application, how healthcare systems will implement future frameworks to regulate their use, especially as newer, more task-specific LLMs are developed, as well as whether these tools can be implemented into more complex tasks across healthcare.
